# Editorial: Advances in genetic engineering strategies for fruit crop breeding, volume II

**DOI:** 10.3389/fpls.2023.1264914

**Published:** 2023-08-16

**Authors:** Tiziana Pandolfini, Bruno Mezzetti, Kevin Folta

**Affiliations:** ^1^ Department of Biotechnology, University of Verona, Verona, Italy; ^2^ Department of Agricultural, Food and Environmental Sciences, Marche Polytechnic University, Ancona, Italy; ^3^ Horticultural Sciences Department, University of Florida, Gainesville, FL, United States

**Keywords:** fruit crops, new genetic technologies, genome editing, cisgenesis, breeding

Breeding of fruit crops is a challenging process that must consider the need to preserve the characteristics of elite cultivars and the urgent need to obtain new cultivars with high resilience and high productivity. Changes in climate and the progressive limits on the use of agrochemicals may require the development of new genetic stocks in relatively short periods of time. Classical and innovative genetic engineering approaches may help achieve these goals. The second volume of this Research Topics aimed to present an update on the genetic tools available for the breeding of fruits crops for new traits. The articles of this Research Topic represent well the opportunities offered by genetic engineering to future fruit crop breeding.

One of the first important issues for breeding is the identification of genes responsible for phenotypic traits of commercial interest and the use of molecular tools to discover the mode of action of these genes. The work of Yuan et al. describes the genetic basis of the white flesh phenotype in strawberry. The comparison by transcriptome and metabolome analysis of two strawberry cultivars, one with red skin and red flesh and the other with red skin and white flesh, allowed to demonstrate that mutation of the transcription factor *Fa*MYB10 hinders its binding to the promoter of the *FaUFGT* gene that encodes for flavonol-O-glucosyltransferases, a key enzyme in the anthocyanin biosynthetic pathway.

These authors also provide evidence that the skin and flesh color of strawberry fruits is related to the number of fully functional copies of *FaMYB10* and that the white flesh phenotype can be obtained with a single mutant allele. This kind of study provides useful information about gene variants that may benefit molecular breeding, and understanding the action mechanisms informs genetic manipulation as well as the fundamental basis of pigment production.

A second interesting issue is how to guide and accelerate the progression of wild species to highly valued cultivars with broad consumer interest. A case study is offered in the article by Cheng et al. The purpose of this work is to use phylogenetic analysis to support the breeding of *Elaeagnus* L. a genus of plants that is commonly grown as ornamental, and it is underutilized as a fruit crop, although its red berries have potential nutritional and perhaps medical properties. Phylogenetic analysis of *Elaeagnus* L species considered both DNA markers in the nuclear genome, such as the ribosomal DNA internal transcribed spacer (ITS), and the chloroplast *matK* gene, and integrated these data with morphological metrics clustering. The results allowed the identification of 10 wild species distributed in different parts of China that possess useful characteristics for fruit crop development. This article demonstrated the power of molecular analysis to provide the theoretical basis for crop domestication and genetic improvement.

The development of genome editing technologies has recently opened a new scenario in plant genetic engineering, allowing specific editing of genes without the persistence of exogenous DNA material. In several herbaceous crops, genome editing has been successfully employed *via Agrobacterium* genetic transformation to generate mutants with improved agronomic traits. In these species, DNA introduced by T-DNA transfer is then eliminated by sexual crossing obtaining non-transgenic mutants. Application of this methodology is problematic in fruit crops because they are mostly clonally propagated to maintain cultivar-specific phenotypic features. Therefore, T-DNA free technologies are needed to apply genome editing in fruit crops.

This Research Topic is taken into account by the articles of Scintilla et al. and Alquezar et al.

Scintilla and colleagues obtained DNA-free edited grapevine plants by applying a single-cell based approach and transfecting CRISPR/Cas editing machinery. They produced protoplasts form embryogenic calli and edited the protoplasts by transfecting them with a ribonucleoprotein complex consisting of the Cas9 protein and an sgRNA for the target gene. From the transfected protoplasts edited non-chimeric plants of two table grape cultivars (*Vitis vinifera* cv. Crimson seedless and Sugraone) were then regenerated. This approach has successfully produced DNA-free edited grapevine plants, but it requires efficient protocols for regenerating plants from protoplasts, which is difficult in most fruit crop species and cultivars.

The approach used in citrus is based on transient expression of nSpCas9 *via Agrobacterium* and recovery of non-transgenic shoots containing the desired mutation. The edited DNA-free shoots represented 17% of the total edited shoots, but the regenerated DNA-free edited shoots were a mosaic of edited/non edited cells. Therefore, this method should incorporate additional selection steps to eliminate chimerism.

Interestingly, in this work, the cytidine deaminase editing system was applied to mutate acetolactate synthase gene to obtain resistance to the herbicide imazapyr (IMZ). The herbicide resistance was then used for selection of edited shoots. This article addresses another important issue for fruit crop engineering, the replacement of antibiotic selection with screening methods based on endogenous genes. The use of IMZ selection can be useful for cisgenic and intragenic modification of fruit crops.

The Research Topics covered in this volume are particularly relevant as they demonstrate that new genetic techniques can bring important benefits to particularly important crops. This information is essential to demonstrate the potential of new technologies to generate consumer and regulatory enthusiasm for application in variety improvement.

Current issues focus on discussion within the European Community on the acceptance of biotech plants obtained using site directed nucleases, like Cas9. These approaches are referred to as New Genetic Technologies (NGT). The European Court of Justice has already banned these technologies but thank to pressure of worldwide acceptance and EU-scientist objections, new proposals are being considered.

The text proposes a legal framework for plants obtained by genetic editing and cisgenesis and for food and feed obtained from them. Different types of NGT plants are identified and it is proposed to regulate plants derived from genetic editing/cisgenesis differently than traditional transgenic (GMO) approaches. This is because genetic modifications are indistinguishable from those obtained by crossbreeding or random mutagenesis. The aim is to maintain a high level of protection of human and animal health and the environment, enable innovation in the agri-food system and contribute to the goals of the European Green Deal and the “Farm to Fork” strategy.

However, the solution currently proposed in the EU is very restrictive. Gene editing is considered acceptable only if additions or deletions are less than 20 nucleotides considerably reducing the potential applications of this technology (Stokstad, 2023). This type of NGT, if the proposal is approved, would still be identified as “GMO” but the notification procedure for human health and environmental risk assessment would be excluded. The verbiage would be replaced by a statement describing the type of NGT modification and be tested directly in the open field without authorization, along with commercial production. The NGT crop would follow normal procedures for commercial development but would be labeled and traced for NGT origin.

However, virtually internationally, these plants remain excluded from the organic certification systems which forbids crops derived from crossbreeding with gene edited and cisgenic plants.

This Research Topic highlights the utility of this technology, so it is appropriate to chart the resistance it still faces before application. Continued work in this area may be valuable in swaying public opinion and lowering regulatory hurdles so that NGT may be employed to benefit the farmer, the food insecure, the environment and the consumer.

## Author contributions

TP: Writing – original draft, Conceptualization, Writing – review & editing. BM: Writing – original draft, Conceptualization, Writing – review & editing. KF: Conceptualization, Writing – original draft, Writing – review & editing.

## References

[B1] Stokstad K. (2023) European Commission proposes loosening rules for gene edited plants. Available at: https://www.science.org/content/article/european-commission-proposes-loosening-rules-gene-edited-plants?utm_source=sfmc&utm_medium=email&utm_campaign=WeeklyLatestNews&utm_content=alert&et_rid=17035139&et_cid=4805948.

